# Discharge within 24 h, transvaginal natural orifice transluminal endoscopic surgery- more suitable for ambulatory surgery in gynecology procedures: a retrospective study

**DOI:** 10.1186/s12905-024-03132-w

**Published:** 2024-05-10

**Authors:** Fangyuan Zhong, Yueyu Dai, Xiaoyan Liao, Wei Cheng, Ying Liu, Yan Liu, Ziru Yan, Yonghong Lin, Xiaoqin Gan

**Affiliations:** 1 Department of Gynecology and Obstetrics, School of Medicine, Chengdu Women’s and Children’s Central Hospital, University of Electronic Science and Technology of China, 1617 Riyue Avenue, Chengdu, Sichuan 610073 China; 2 The Second Affiliated Hospital of Chongqing Medical University, Chongqing, 400042 China

**Keywords:** VNOTES, Laparo-endoscopic single-site surgery, Ambulatory surgery, Gynecology procedures

## Abstract

**Background:**

Natural orifice transluminal endoscopic surgery (NOTES) is an achievement in the field of minimally invasive surgery. However, the vantage point of vaginal natural orifice transluminal endoscopic surgery (vNOTES) in gynecologicalprocedures remains unclear. The main purpose of this study was to compare vNOTES with laparo-endoscopic single-site surgery, and to determine which procedure is more suitable for ambulatory surgery in gynecologic procedures.

**Methods:**

This retrospective observational study was conducted at the Department of Gynecology, Chengdu Women’s and Children’s Central Hospital. The 207 enrolled patients had accepted vNOTES and laparo-endoscopic single-site surgery in gynecology procedures from February 2021 to March 2022. Surgically relevant information regarding patients who underwent ambulatory surgery was collected, and 64 females underwent vNOTES.

**Results:**

Multiple outcomes were analyzed in 207 patients. The Wilcoxon Rank-Sum test showed that there were statistically significant differences between the vNOTES and laparo-endoscopic single-site surgery groups in terms of postoperative pain score (0 vs. 1 scores, *p* = 0.026), duration of anesthesia (90 vs. 101 min, *p* = 0.025), surgery time (65 vs. 80 min, *p* = 0.015), estimated blood loss (20 vs. 40 mL, *p* < 0.001), and intestinal exhaustion time (12.20 vs. 17.14 h, *p* < 0.001). Treatment with vNOTES resulted in convenience, both with respect to time savings and hemorrhage volume in surgery and with respect to the quality of the prognosis.

**Conclusion:**

These comprehensive data reveal the capacity of vNOTES to increase surgical efficiency. vNOTES in gynecological procedures may demonstrate sufficient feasibility and provide a new medical strategy compared with laparo-endoscopic single-site surgery for ambulatory surgery in gynecological procedures.

## Background

NOTES focuses on the natural orifices of the body, known as the mouth, anus, umbilicus, urethra, and vagina [1]. It was first performed in 2004 in a porcine model by researchers at Johns Hopkins University [2]. Since 2007, as a less invasive procedure, vNOTES has been performed in gynecology practice for the surgical treatment of ovarian cysts, intramural uterine fibroids, ectopic pregnancies, adnexal masses, and pelvic organ prolapse [3–6]. Minimally invasive surgeries such as vNOTES are frequently performed [7, 8].

Laparoscopic skills allow minimally invasive surgical procedures to be performed in many gynecological procedures. Laparo-endoscopic single-site surgery has advantages over conventional multiple-port laparoscopic practice, on behave of cosmetic appearance, decreased port site pain, and less duration of surgeries [9]. Advances in technology and laparoscopic techniques have enabled a shift in gynecological surgery from inpatient to more time-efficient settings. Ambulatory surgery, also known as same-day discharge, refers to an operation in which the patient is admitted and discharged within 24 h; in special cases, it should not exceed 48 h [10]. The growing relevance of the Enhanced Recovery after Surgery (ERAS) concept requires surgeons to continuously refine their surgical skills to ensure less surgery-related damage and pain, shorter hospital stays, and earlier return to normal life [11, 12]. After the concept of ambulatory surgery was first proposed in 1909, it has rapidly developed in patients undergoing laparoscopic procedures for benign indications. Ambulatory surgery has been demonstrated to be safe, money-saving, and acceptable to patients [13, 14].

This study aimed to determine which vNOTES and laparo-endoscopic single-site surgery are more suitable for Ambulatory surgery in gynecological practice.

## Methods

This study was conducted at the Department of Gynecology, Chengdu Women’s and Children’s Central Hospital and School of Medicine, University of Electronic Science and Technology. Patients were enrolled if they underwent laparo-endoscopic single-site surgery and vNOTES between February 1, 2021, and March 31, 2022, with one of four attending surgeons who were members of the Department of Gynecology at Chengdu Women’s and Children’s Central Hospital. Patients with poorly controlled medical comorbidities were not eligible for same-day discharge. Patients with advanced (≥ 65 years), congestive heart failure, hepatic disease, history of cerebrovascular accidents, and poorly managed sleep apnea were excluded from the study. Patients were excluded if the pathological analysis of a biopsy revealed malignancy. Patients who required conversion to laparotomy were excluded from the study.

All patients were treated during the course of surgery with ERAS. Patients received preoperative education, urinary catheters and drainage tubes were not placed during surgery, and patients were encouraged to eat and get out of bed as soon as possible after surgery to achieve rapid recovery.

All data were extracted from the patients’ medical records. Four gynecologic surgeons performed vNOTES or laparo-endoscopic single-site (LESS) surgery in the patients included in this study. Laparoscopic instrumentation was performed according to surgeon preference. Complications that occurred within 30 days of surgery were abstracted and categorized by type. The clinical characteristics analyzed included age, body mass index, prior medical or surgical history; postoperative complications such as mortality, unplanned reoperation, delayed discharge, readmission, duration of anesthesia, execution time of surgery, estimated blood loss, postoperative pain score, infection (urinary tract infection, pneumonia), nausea and vomiting, venous thromboembolism, and patient satisfaction within 30 days. Postoperative bleeding that required readmission or blood transfusion was classified as a hemorrhagic complication.

SPSS 22.0 statistical software was used for statistical analysis. The measurement data were expressed as mean and median such as age, BMI. The measurement data of non-normal distribution were described by the median. The Wilcoxon Rank-Sum test was used for comparisons between two groups. All statistical analyses in this study were conducted by two-sided tests, and *P* < 0.05 was indicative of statistical significance.

## Results

Multiple outcomes of 207 patients were analyzed; of these, 39 patients had uterine fibroids (2 vNOTES, 37 LESS), 51 underwent tubal surgery for ectopic pregnancy (36 vNOTES, 15 LESS), six patients were treated for the presence of an ovarian mass (23 vNOTES, 40 LESS), 20 underwent tubal sterilization (20 LESS), and fivepatients underwent hysterectomy (5 LESS) (due to endometrioma, uterine myoma, and CIN endometrial disease). In addition, 29 patients underwent surgery for endometriosis, cervical intraepithelial lesions, endometrial disease, or diseases. After completion of the Wilcoxon Rank-Sum test, especially for the median, there were statistically significant differences between the vNOTES and laparo-endoscopic single-site surgery groups.

As shown in Table 1, BMI, time spent getting out of bed, urination, and bowel movement were not significantly different between the two groups. Only 3 patients with unplanned reoperation and readmission were included in both the vNOTES and LESS groups. Two of them underwent tubal fenestration surgery but unfortunately had persistent ectopic pregnancy and reoperation for salpingectomy. NOTES assisted vaginal myomectomy was performed to address a 7 cm intramural uterine fibroid located in the posterior uterine wall. The procedure lasted 107 min. However, due to postoperative hematoma formation, the patient required readmission for further treatment. Nine patients (1 vNOTES, 8 LESS) had delayed discharge. One patient in the vNOTES group refused to be discharged the day after surgery for personal reasons. In the LESS group, two patients had fever after surgery, four had hysterectomies, and one had severe anemia and have to stay in the hospital for a blood transfusion. Four patients had delayed discharge due to intestinal exhaust. None of the patients had venous thromboembolism.

As shown in Table 2, the postoperative pain score (0 vs. 1, *p* = 0.026), duration of anesthesia (90 vs. 101 min, *p* = 0.025), duration of surgery (65 vs. 80 min, *p* = 0.015), estimated blood loss (20 vs. 40 mL, *p* < 0.001), and intestinal exhaust time (12.20 vs. 17.14 h, *p* < 0.001) were significantly different. Some patients may require pain relief through oral pain relievers (such as ibuprofen) or intramuscular injections (e.g., pethidine), particularly in the evening after surgery. In this study, the postoperative pain score (vNOTES, 0 vs. LESS, 1) indicated that vNOTES may result in less pain in patients undergoing surgery. The overall duration of the surgical procedures ranged from 55 to 215 min, indicating variability based on factors such as the size and complexity of the mass being treated. In this study, vNOTES procedures were used to treat various pathologies, with a mean surgery duration of approximately 65 min, which is comparable to that of LESS procedures (80 min). Surgeries involving endometrioma and dense pelvic adhesions tended to increase operative time. These studies were conducted primarily using the LESS approach. The comparison between vNOTES and LESS procedures revealed that vNOTES surgery, with an anesthesia duration of 90 min, is more time efficient than LESS surgery. LESS surgery requires 101 min of anesthesia. The comparison of estimated blood loss between vNOTES and LESS highlights another advantage of vNOTES. vNOTES resulted in only 20 mL of blood loss compared with 40 mL with LESS.

**Table 1:** Perioperative characteristics of the gynecology procedures

Parameter	vNOTES *N* = 64	Laparoscope *N* = 143	*p* Values
Age	33.03	36.52	0.005
BMI	22.94	22.64	0.554
Got out of bed and move(min)	181.61	188.10	0.515
Urination(min)	184.11	193.52	0.380
Bowel movement(h)	47.11	49.32	0.221

Values are expressed as means, medians, and ranges for continuous and absolute numbers

**Table 2:** Perioperative characteristics of gynecology procedures

Parameter	vNOTES *N* = 64	Laparoscope *N* = 143	*p* values
Postoperative Pain Score	0	1	0.026
Execution Time of Surgery (min)	65	80	0.015
Duration Period of Anesthetization (min)	90	101	0.025
Estimated Blood Loss (mL)	20	40	< 0.001
Intestinal Exhaust (h)	12	17	< 0.001

## Discussions

Ambulatory surgery offers significant advantages by enhancing patient convenience and optimizing the utilization of hospital resources. Additionally, it contributes to substantial cost savings in healthcare and reduces the incidence of nosocomial infections [15]. Advanced laparoscopic gynecologic surgery is safe and effective in ambulatory surgery centers. As the foundations of laparoscopic surgery in gynecology have expanded, it has become the standard of care. Moreover, the skills of individual surgeons and the availability of advanced instrumentation continue to improve. This minimally invasive approach offers the advantage of minimal abdominal wall trauma.

Minimally invasive surgeries such as vNOTES are frequently performed [7, 8]. Baekelandt et al. reported the feasibility of NOTES for treating uterine fibroids in eight cases. All patients were successfully treated without complications and required conversion to standard laparoscopy. Based on their findings, they concluded that vNOTES could serve as a minimally invasive approach for the treatment of uterine fibroids [4]. Another area in which NOTES is useful is the treatment of ectopic pregnancies. Baekelandt et al. conducted a study focusing on the treatment of pregnancies with unknown locations in 15 patients [5]. Their approach involved initiating transvaginal hydro-laparoscopy and subsequently performing salpingectomies using the vNOTES technique in 12 patients, with successful outcomes. Kaya et al. reported that vNOTES is a feasible technique for obese women who require a hysterectomy. It provides favorable outcomes, including a shorter surgery duration and postoperative hospitalization. Additionally, patients experienced lower pain scores [6]. Huang had a study of 1147 patients underwent vNOTES of adnexal surgery, myomectomy, hysterectomy, pelvic floor reconstruction surgery, and malignant tumor surgery, and found that the application of vNOTES is safe and feasible for most gynecological surgeries [16].

The advantage of magnifying the surgical area with optical systems is that it decreases the umbilical or port-site hernia rates. The NOTES procedure has better outcomes than conventional laparoscopic and open surgeries [9, 17]. Potential advantages of vNOTES compared to traditional laparoscopic and robotic approaches include the potential for less pain, decreased operative time, improved cosmesis, and decreased risks [18, 19]. Karakaş recently reported the compare of vNOTES with conventional laparoscopy regarding pre-/intra-/postoperative outcomes. In the study, the women who were operated on for emergency indications such as ectopic pregnancy, ovarian torsion, ovarian cyst rupture and acute abdominal pain were evaluated, suggesting that vNOTES could serve as an alternative to conventional laparoscopy. They offer advantages such as shorter surgery duration, lower postoperative pain scores, shorter hospital stays, and better cosmetic outcomes [20], mirroring the results of the current study. Cihan Kaya et al. reported that hysterectomy performed using vNOTES had a shorter mean operation duration compared to both standard laparoscopy and laparotomy [21]. They also compared the results of conventional laparoscopic and vNOTES techniques for the treatment of benign adnexal pathologies. The study consisted of 114 patients for oophorectomy, ovarian cystectomy, or ectopic pregnancies, showed that the duration of surgery was significantly shorter in the vNOTES group compared to the conventional laparoscopy group, postoperative hospital stay was significantly shorter in the vNOTES group, Postoperative 6th- and 24th-hour VAS pain scores were significantly lower in the vNOTES group [22].

Recent studies also have assessed the feasibility of NOTES procedures. These studies found that NOTES procedures were associated with shorter hospital stays, shorter duration of surgery, and reduced total use of analgesics compared to LESS [23]. vNOTES was equally safe and effective for ovarian cystectomy compared to LESS. vNOTES aligned with the concept of the day-care procedure due to its reduced postoperative pain, shorter exhaust time, and absence of scarring. However, surgeons should conduct a comprehensive preoperative evaluation and exclude patients suspected to have severe pelvic adhesions [24]. vNOTES can shorten the exhaust time and duration of hospitalization, reduce postoperative pain, and avoid surface surgical scars in tubal pregnancy surgeries, consistent with the ERAS concept [25].

In Karakaş’ study [20], vNOTES group had lower VAS scores after 6 h than the conventional laparoscopy group. In our study, the postoperative pain score (vNOTES, 0 vs. LESS, 1) indicated that vNOTES may result in less pain in patients undergoing surgery. However, the necessity for postoperative analgesia depends on an individual patient’s pain tolerance, leading to highly subjective data. The vagina is relatively less sensitive than the abdominal nerves, resulting in less pain when an incision is made in the posterior fornix of the Patients who underwent vNOTES, as opposed to LESS, reported less pain and required fewer painkillers. This advantage allows patients to mobilize sooner after surgery, thereby facilitating recovery of intestinal function.

The surgical execution time (vNOTES 65 min vs. LESS 80 min) suggests that the vNOTES procedure is more time efficient, which is the same with Karakaş’ study [20]. vNOTES reducing the duration of surgery, the day surgery mode becomes more efficient, facilitating the effective utilization of surgeons, nursing staff, and operating room resources.

This time-saving aspect of vNOTES not only reduces the duration of anesthesia but also minimizes the number of anesthetic drugs administered. Consequently, vNOTES can effectively alleviate postoperative gastrointestinal reactions such as nausea and vomiting as well as mitigate discomforts such as dizziness and headaches. Moreover, vNOTES facilitated swift restoration of gastrointestinal function by minimizing the inhibition of intestinal peristalsis. In the present study, patients undergoing vNOTES exhibited shorter intervals to intestinal exhaust, leading to fewer instances of delayed discharge than those undergoing LESS. This observation underscores the advantages of vNOTES in promoting quicker postoperative recovery and optimizing patient outcomes.

The lower blood loss in patients treated with vNOTES contributed to the expedited postoperative recovery and faster restoration of normal vital signs. Additionally, it reduces the likelihood of postoperative blood transfusion. Based on clinical experience, researchers have observed that patients with higher postoperative hemoglobin levels have a decreased risk of developing complications such as postoperative infections. Therefore, minimizing blood loss during surgery, as achieved with vNOTES, may help mitigate these potential complications and improve the overall patient outcomes.

This study has several limitations. The retrospective nature of the study may have been constrained by inherent selection bias. Additionally, limitations related to the availability of medical records and the possibility of transcription errors could affect the accuracy. Moreover, the number of postoperative complications may be underreported as patients may have sought care at their physician’s office or a different hospital. Furthermore, while much of the focus has been on outcomes and costs, it is crucial to include discussions that compare measures of patient experience. This encompasses experiences from the waiting room to the care received pre- and post-operatively.

These comprehensive data revealed the capacity of vNOTES to enhance surgical efficiency in ambulatory surgery. Treatment with vNOTES not only saves time and reduces hemorrhage volume during surgery but also improves the quality of prognosis. Similar findings have been reported in other studies, suggesting that vNOTES could serve as an alternative to conventional laparoscopy. They offer advantages such as shorter surgery duration, lower postoperative pain scores, shorter hospital stays, and better cosmetic outcomes [20], mirroring the results of the current study. vNOTES procedures in gynecology have demonstrated sufficient feasibility and provide a new medical strategy compared with laparo-endoscopic single-site surgery in ambulatory surgery in the ERAS mode.

In conclusion, vNOTES procedures showed promising results in treating various gynecological diseases and may be more suitable than LESS procedures in ambulatory surgery centers. However, further investigation is warranted to validate these findings and achieve better surgical outcomes in the near future.

## Data Availability

The data that support the findings of this study are available on request from the corresponding author. The data are not publicly available due to privacy restricions.
